# Supplementary material for: Phenotypes and Genotypes of Inherited Disorders of Biogenic Amine Neurotransmitter Metabolism
Source: Genes (Basel). 2023 Jan 19;14(2):263. doi: 10.3390/genes14020263 (PMC9957200; doi:10.3390/genes14020263)
Supplement: Supplementary file 1 [file genes-14-00263-s001.zip › File S1.pdf]

File S1: Variants recorded as pathogenetic and associated with biogenic amine defects in the BioPKU (PNDdb http://www.biopku.org, accessed on 7 October 2022) and ClinVar (https://www.ncbi.nlm.nih.gov/clinvar/, accessed on 7 October 2022) databases (Supplement S1).

The pathogenetic ClinVar variants with conflicting interpretations, without assertion criteria of pathogenicity and structural variants with a length >1 Kb, involving multiple genes, and for which the clinical phenotype does not match with biogenic amine disorder were filtered out.

HGVS Variants' nomenclature format was revised through Mutalyzer sequence variation name checker (www.lovd.nl/mutalyzer, accessed on 7 October 2022). LitVar2 (https://www.ncbi.nlm.nih.gov/research/litvar2/, accessed on 7 October 2022) was used for most variants' references.
